# Psychotherapy by Telephone or Internet in Austria and Germany Which CBT Psychotherapists Rate It more Comparable to Face-to-Face Psychotherapy in Personal Contact and Have more Positive Actual Experiences Compared to Previous Expectations?

**DOI:** 10.3390/ijerph17217756

**Published:** 2020-10-23

**Authors:** Nicole Korecka, Rafael Rabenstein, Christoph Pieh, Peter Stippl, Antonia Barke, Bettina Doering, Katharina Gossmann, Elke Humer, Thomas Probst

**Affiliations:** 1 Department for Psychotherapy and Biopsychosocial Health, Danube University Krems, 3500 Krems, Austria; nicole.korecka@edu.donau-uni.ac.at (N.K.); rafael.rabenstein@donau-uni.ac.at (R.R.); christoph.pieh@donau-uni.ac.at (C.P.); elke.humer@donau-uni.ac.at (E.H.); 2 Austrian Federal Association for Psychotherapy, Löwengasse 3, 1030 Vienna, Austria; oebvp.stippl@psychotherapie.at; 3 Clinical and Biological Psychology, Catholic University of Eichstätt-Ingolstadt, Ostenstraße 26, 85072 Eichstätt, Germany; antonia.barke@ku.de (A.B.); bettina.doering@ku.de (B.D.); katharina.gossmann@ku.de (K.G.)

**Keywords:** psychotherapists, remote psychotherapy, telephone, internet, experiences, expectations, comparability, COVID-19

## Abstract

*Objectives*: COVID-19 has led to changes in the provision in mental health services. The current study investigated influencing factors on: (i) the comparability of psychotherapy via internet/telephone with psychotherapy in face-to-face contact as well as (ii) the actual experience with psychotherapy via internet/telephone compared to respective prior expectations in CBT therapists. *Methods*: A quantitative cross-sectional study was conducted in the form of an online survey. The research samples, registered cognitive-behavioral therapy (CBT) psychotherapists in Austria and Germany, were contacted by e-mail. *Results*: One hundred and ninety CBT therapists were analyzed in this study. The total number of patients treated via telephone/internet is a decisive factor for the subjective evaluation of the comparability of psychotherapy via telephone/internet and psychotherapy in personal contact. This factor also influences the extent (positive/negative) of the assessment of the actual experience with psychotherapy via internet/telephone compared to previous expectations. Neither age nor gender were associated with comparability of psychotherapy via internet/telephone with psychotherapy in face-to-face contact or the actual experience with psychotherapy via internet/telephone compared to respective prior expectations. *Conclusions*: Implications of the results are that attitudes towards remote psychotherapy might be increased in CBT therapists when they treat more patients remotely and experiences with remote psychotherapies should be included in psychotherapy training.

## 1. Introduction

The novel corona virus (COVID-19) keeps people around the world concerned. It has far-reaching consequences, not only for the economy but also for the health system. People are restricted in their freedom as well as in their professional and family life by the measures to contain the corona virus. These restrictions also have an impact on the physical and mental health of people [1], including those at the forefront of healthcare [2]. Consequently, an increase in mental health care utilization can be expected in the near future and provision of safe and efficient mental health services is urgently needed [3]. Because psychotherapy is an essential part of the health care system, psychotherapists have to handle the massive consequences of COVID-19 and adapt to the current situation in order to be able to continue to provide care for patients. Due to the currently valid protective measures, a change to psychotherapy via telephone and internet takes currently place since face-to-face psychotherapies in personal contacts are either not possible or only with several restrictions [4]. Psychotherapy via telephone or internet is related to telemedicine, which is defined as a resource that uses technology and associated services to deal with health and well-being, is available over distance and provides care, nursing and support to people in this context [5]. The results of a study in Germany, the Czech Republic and Slovakia indicate that psychotherapy in personal contact is nevertheless the most common treatment modality during COVID-19 and that changes in the provision of psychotherapy varies between countries [6]. In Austria, for example, the number of patients treated in face-to-face contact during the lockdown decreased and the number of patients treated via the internet or telephone increased. Nevertheless, the results of the Austrian study point to an undersupply of psychotherapy in the COVID-19 lockdown [7].

Mental health care providers and patients can gain important advantages through technology-based treatments, for example, easier access and similar effectiveness compared to face-to-face therapy [8,9]. Although telemedicine has already been implemented in various clinical settings for a wide range of target groups [10], for example in England where in some programs, such as the Improving Access to Psychological Therapies (IAPT) services, remote treatment has been integrated [11]. Nevertheless, many healthcare providers have made limited use of telemedicine and thus have little experience of its utilization in the past [4,12,13]. As a help, the American Psychiatric Association and the American Telemedicine Association [14] have provided consensus guidelines for becoming familiar with telemedicine. A meta-analysis of several studies shows that the barriers to implementing telemedicine are mainly technically challenged staff (11%), followed by resistance to change (8%), costs (8%), reimbursement (5%), age of the patient (5%) and level of education of the patient (5%) [15]. A Syrian study, for example, in which 52 humanitarian health care institutions participated, reported that the majority of providers have no experience with telemedicine. As barriers to such services, respondents cited cultural (68%), financial (84%) and technical (80%) factors. Overall, the institutions were open to the use of tele-mental health services [16]. A study in Israel also found that the willingness to use telemedicine is influenced by attitudes towards it, attitudes towards the doctor-patient relationship and the degree of technology fear [17]. Regarding provision of mental health care via videoconferencing (TMH-V: tele-mental health conducted via videoconferencing), a review showed that providers have a positive attitude towards this technology overall. Respective attitudes are supposed to be related to the earlier TMH-V experience so that acceptance of this form of treatment increases with usage [13]. An Austrian study, in which 1547 psychotherapists with different theoretical orientations (psychodynamic, humanistic, systemic, behavioral) were surveyed, showed that psychotherapists rated psychotherapy via telephone or internet as not fully comparable to psychotherapy in personal contact [18]. However, psychotherapy via the internet was perceived by psychotherapists to be more comparable to psychotherapy in personal contact than psychotherapy via telephone. With regard to the telephone (but not the internet) the rating on the ability to treat patients equally as in personal contact was affected by the theoretical background. More specifically, behavioral therapists (N = 101, M = 41.7, SD = 24.97) scored lowest, followed by systemic (N = 233, M = 48.6, SD = 26.57) therapists. Humanistic therapists (N = 468, M = 51.2, SD = 25.64) and psychodynamic therapists scored highest (N = 205, M = 53.9, SD = 25.78), differing significantly from behavioral therapists (p ≤ 0.005). Among therapeutic orientations, the actual experiences with psychotherapy via telephone and internet were better than previously expected. However, for psychotherapy via telephone differences concerning the theoretical orientation were observed. Behavioral therapists (N = 73, M = 60.6, SD = 21.87) scored lowest, followed by systemic therapists (N = 177, M = 60.8, SD = 20.64) and humanistic therapists (N = 370, M = 64.8, SD = 17.48). Psychodynamic therapists (N = 157, M = 68.3, SD = 18.98) had the highest score for actual experiences with psychotherapy via telephone in relation to their previous expectations, differing significantly from behavioral and systemic therapists (*p* ≤ 0.027) [18]. Gilmore and Ward-Ciesielski [19], in their research on the use of telemedicine in psychotherapy, were able to show that more experienced therapists have a more positive attitude towards telemedicine, as well as younger ones compared to older providers. The results of an actual research suggest that psychotherapists attitudes toward remote psychotherapy are influenced by their past experiences, such as psychotherapy modality, clinical experience, and previous online treatment experience [20]. An earlier study by Hanson et al. [21] found that the actual use of telemedicine improved attitudes toward telemedicine of the health care providers who used telemedicine for the first time. Most of the study results indicate a positive correlation between increasing experience and the attitude towards telemedicine [13]. In a qualitative study on clinical work by telephone, the respondents reported mainly positive experiences. The actual experience often varied from previous expectations and attitudes towards telemedicine [22]. In view of the novel coronavirus, further investigations could show that the application of technology is already strongly integrated in the implementation of remote treatment by psychotherapists. Furthermore, the psychotherapists proved to be more confident and comfortable in the actual current experience and implementation of remote treatment than previously expected [23].

Even before the appearance of the new corona virus, treatment formats such as telephone and internet-based psychotherapy were offered and carried out as part of psychotherapy in various countries, based on research findings pointing to comparable effectiveness to face-to-face psychotherapy [24,25], especially for CBT methods [26,27]. Many therapists used the telephone in their psychotherapeutic work mainly for administrative tasks and formal matters. The research results of a German study by Eichenberg and Kinzle [28] indicated that almost a quarter of the respondents also received treatment-related calls and that by giving out their mobile phone number they gave patients the opportunity to contact them in particularly difficult situations. About 10% of the respondents offered, in addition to face-to-face psychotherapy, psychotherapeutic internet services to support therapeutic processes (69.6%), as a partial component of therapeutic interventions (52.2%) and to provide follow-up care (34.8%). Similar to the results of this German research, a study by Cipolletta and Mocellin [29] reported that 18.3% of the interviewed Italian psychologists had experience with online therapy. The majority of the respondents (62.6%) additionally supported therapy in the form of online contact, although some reservations existed concerning the implementation of the diagnostic-therapeutic process. The study also demonstrated that there is not enough clarity in this context in terms of ethical and legal issues. In contrast, 37.2% of Portuguese psychologists stated in another study that they were familiar with internet interventions [30]. The main benefits of internet interventions were identified as accessibility (79.9%), comfort (45.7%) and cost efficiency (45.5%). Ethical concerns (40.7%), information and communication technology illiteracy (43.2%) and attitudes towards Internet interventions (37%) were identified as the main limitations. Overall, the results of Mendes-Santos et al. indicate a higher acceptance of mixed treatment interventions (62.9%) compared to independent internet interventions (18.6%). A recent study came to similar conclusions: The lack of knowledge and trainings turned out to be the main factor for a rather negative/neutral attitude (37%) towards internet interventions [31]. Results from a Norwegian survey confirmed this aspect [32]. In contrast, a German study showed that factors such as knowledge about internet interventions, frequency of internet use, age and gender of psychotherapists have no significant impact on the attitude towards internet interventions [33]. Yet, the research team was able to identify a correlation between increasing age and reduced belief in the effectiveness of internet interventions. Another study, in which psychologists were interviewed, revealed age as a significant influencing factor for using telemedicine as a complement to personal contact. According to these findings, psychologists under the age of 45 have a higher probability of using telemedicine interventions than those over 45 [34].

Regarding psychotherapy in Austria, research has shown that there is also a lack of clarity and knowledge in the implementation of technology-supported treatments [35]. Only 12 to 13% of the surveyed psychotherapists used computers in the context of psychotherapy for video conferencing or sharing materials (e.g., videos or book chapters) with patients. In Austria, the official guideline on psychotherapy via internet rejects internet-based psychotherapy [36]. However, in the actual special situation around COVID-19 most health insurance companies in Austria started to cover costs also for psychotherapy via internet and telephone. In Germany, psychotherapists can conduct up to 20% of their overall treatments per quarter via internet with an upper limit of 20% of patients per quarter receiving internet psychotherapy as only treatment format. Some psychotherapeutic services, however, such as the initial interview or diagnostics are only covered when conducted face-to-face [37]. Psychotherapy via telephone is not possible, however a very limited number of phone contacts are reimbursed as psychotherapeutic contacts. In the COVID-19 crisis, soon after the start of the lockdown up to 1 June 2020, this previous limit concerning the amount of internet therapy was suspended and additional services were reimbursed when conducted remotely.

Based on previous studies, this paper aims to examine the experiences of Austrian and German cognitive-behavioral therapists with remote psychotherapy and the factors influencing them in the early weeks of the COVID-19 lockdown. The lockdown in Austria began on 16th of March 2020, in Germany on 23rd of March 2020 [38]. The following research questions (RQ) will be investigated:

RQ1: For CBT psychotherapists using telephone for psychotherapy either before or in the COVID-19 lockdown: Which factors influence the subjective assessment of the perceived comparability (less/more) of psychotherapy via telephone and psychotherapy in personal contact? We had no specific hypothesis here and this is an explorative research question in times of COVID-19.
RQ2: For CBT psychotherapists using internet for psychotherapy either before or in the COVID-19 lockdown: Which are the influencing factors for the subjective assessment of the perceived comparability (less/more) of psychotherapy via internet and psychotherapy in personal contact? Based on the studies showing that older people have less trust in the effectiveness of internet interventions, we hypothesized that older psychotherapists experience psychotherapy via the internet less comparably than younger psychotherapists. Furthermore, it can be assumed that those therapists who have more experience with internet interventions experience this form of treatment more comparably to psychotherapy in personal contact.
RQ3: For CBT psychotherapists who did not use telephone for psychotherapy before COVID-19 and started to use telephone for psychotherapy during COVID-19 lockdown: Which psychotherapists report more negative/positive actual experiences of using telephone for psychotherapy compared to their previous expectations regarding to the factors age, sex, sum of patients? We had no specific hypothesis here.
RQ4: For CBT psychotherapists who did not use internet for psychotherapy before COVID-19 and started to use internet for psychotherapy during COVID-19 lockdown: Which psychotherapists report more negative/positive actual experiences of using the internet for psychotherapy compared to their previous expectations regarding to the factors age, sex, sum of patients? Based on the literature, we hypothesized that younger psychotherapists evaluate the actual experience more positively (compared to previous expectations) than older psychotherapists.
RQ5: For psychotherapists using internet for psychotherapy either before or in the COVID-19 lockdown: Do their ratings on “knowledge/feeling informed” about using internet for psychotherapy correlate with the ratings regarding comparability of psychotherapy via internet and psychotherapy in personal contact? Based on the literature showing that concerns about ethical and legal issues have a negative influence, we proposed this hypothesis: CBT-psychotherapists who feel less informed about legal issues experience psychotherapy via internet as less comparable to face-to-face psychotherapy in personal contact. Moreover, we evaluated whether the ratings of “knowledge/feeling informed” differ between psychotherapists not treating patients via internet and psychotherapists treating at least one patient via internet. Again, we hypothesized that the ratings of “knowledge/feeling informed” are higher in those providing treatment via the internet.

## 2. Materials and Methods 

### 2.1. Study Design

To investigate our research questions, a quantitative cross-sectional study was conducted in the form of an online survey using the platform REDCap. The research sample, registered psychotherapists in Austria and Germany, was contacted by e-mail (Austria: around 6000 licensed psychotherapists were contacted on 24 March 2020; Germany: around 1700 licensed psychotherapists were contacted on 19 May 2020). More details about the Austrian survey can be found in Humer, Stippl et al. [18], and Probst et al. [7,39]. More details about the German survey can be found in Humer, Pieh et al. [6].

In Austria, COVID-19 lockdown measures started on 16th of March 2020. In Germany, COVID-19 lockdown measures started on 23rd of March 2020. In Austria, e-mail addresses were exported from the official list of psychotherapists (around 9000 registered psychotherapists in March 2020). In Germany, e-mail addresses were gathered from the homepages of different regional and national associations for licensed psychotherapists. According to the consent given by the licensed psychotherapists these associations offer publicly available contact information of their relevant members. In total, we used the contact information gathered from four different psychotherapeutic associations in Germany. For the current study, only the CBT psychotherapists were analyzed.

### 2.2. Participants

In total, 237 CBT therapists (151 in Austria and 86 in Germany) participated in the online survey. To address the RQs, only those therapists were analyzed who answered yes to the questions whether they practiced in the months before COVID-19 and whether they continued to practice in the COVID-19 situation (to have CBT therapists experienced in usual psychotherapy before COVID-19 and changed psychotherapy during COVID-19). This sample for the current study comprised 190 CBT therapists (120 in Austria and 70 in Germany). Among the surveyed psychotherapists, 78.1% were female, which corresponded to the population of psychotherapists in Austria (73.32% as of October 2019). The sample descriptions and the comparisons between in- and excluded CBT therapists are shown in Table 1. There were no significant differences between the included and excluded samples.

### 2.3. Measures

Psychotherapists reported their age, gender, the number of patients treated on average per week via telephone and the number of patients treated on average per week via internet. The number of patients treated on average per week was asked for the months before COVID-19 as well as during COVID-19. We analyzed the sum of patients treated with telephone/internet before/during COVID-19. In addition, the following slider items were used. 

### 2.4. Comparability 

To measure the subjective assessment of the perceived comparability of the implementation of psychotherapy via internet/telephone to psychotherapy in face-to-face contact, we used a slider from “does not apply at all” to “fully applies”, in values from 0 to 100. These questions were only posed to psychotherapists who treated at least one patient with telephone/internet either before or during COVID-19.

### 2.5. Actual Experiences

To measure the actual experiences (more positive/negative) compared to previous expectations in relation to the use of internet or telephone in psychotherapy, we used a slider from “more negative than expected” to “ more positive than expected”, in values from 0 to 100. These questions were only posed to psychotherapists who started to treat patients via telephone/internet in COVID-19, since a previous study has shown that the “new users of telehealth” show the most marked changes of attitudes [21]. 

### 2.6. Knowledge about Legal and Regulatory Issues Concerning Psychotherapy via Internet 

Regarding the construct “feeling informed”, this variable was only requested from the CBT therapists licensed in Austria, since their situation for internet-based psychotherapy was special during COVID-19 in that the Austrian guideline for internet and psychotherapy rejects internet-based psychotherapy, but the health insurances started to cover costs for it during the COVID-19 situation. We provided information to the respondents by sharing the link with the internet Guidelines for Psychotherapists of the Federal Ministry of Social Affairs, Health and Communication [36] and a text fragment of the responsible department of the Ministry of Health on the current situation around COVID-19. As a third source of information we shared an update of the Austrian Federal Association for Psychotherapy (ÖBVP) about the current situation of coronavirus and provision of psychotherapy during it. Subsequently, we asked how well-informed the participants felt about the use of internet in psychotherapy. Again, an answer could be marked on a scale from “not at all” to “very good”, in values from 0 to 100. 

### 2.7. Statistics

For statistical analysis, the software program IBM SPSS Statistics for Windows Version 26.0 (IBM Corp, Armonk, NY) was used. To answer the research questions, we calculated Pearson correlations and a t-test. All statistical tests were performed two-tailed and the significance level was set to *p* < 0.05.

## 3. Results

Results are summarized in Table 2.

Results for RQ1: The only significant correlation emerged between the number of patients being treated via telephone and the subjective assessment of the comparability of psychotherapy via telephone with psychotherapy in personal contact (*r* = 0.240, *p* = 0.003, *n* = 151). 

Results for RQ2: Comparability of psychotherapy via the internet with psychotherapy in personal contact was significantly correlated only with the number of patients treated by internet, *r* = 0.198, *p* = 0.019, *n* = 141. 

Results for RQ3: Psychotherapists who began to treat a higher number of patients via telephone during COVID-19 had more positive actual experiences with psychotherapy via telephone compared to previous expectations, *r* = 0.238, *p* = 0.010, *n* = 116. Age and gender had no influence on the rating of how negative/positive actual experiences compared to previous expectations for psychotherapy via telephone. 

Results for RQ4: Psychotherapists who began to treat a higher number of patients via internet during COVID-19 had more positive actual experiences with psychotherapy via internet compared to previous expectations (positive/negative), *r* = 0.341, *p* < 0.001, *n* = 122. 

Results for RQ5: Those psychotherapists from Austria which feel more/better informed reported higher comparability between psychotherapy via internet and psychotherapies in personal contact, *r* = 0.242, *p* = 0.025, *n* = 85. In addition, psychotherapists from Austria who did not treat patients via internet (*n* = 35; *M* = 74.80; *SD* = 28.61) did not differ in ratings of feeling informed compared to psychotherapists from Austria treating at least 1 patient via internet (*n* = 85; *M* = 72.81; *SD* = 25.23), *T* (118) = 0.377; *p* = 0.707.

## 4. Conclusions

This study evaluated which CBT psychotherapists in Austria and Germany experience remote psychotherapy (telephone, internet) more or less comparable to face-to-face psychotherapy in personal contact. Additionally, we investigated for which CBT psychotherapists the actual experiences of remote psychotherapy (telephone, internet) were more negative/positive compared to previous expectations.

Regarding RQ1, we were able to show a positive correlation between the total number of patients (using the telephone) and the perceived comparability of psychotherapy via telephone to psychotherapy in personal contact. Age and gender did not influence the subjective assessment of the comparability of psychotherapy via telephone and psychotherapy in personal contact.

Concerning RQ2, the hypothesis that psychotherapists who have more experience with psychotherapy via the internet perceive it to be more comparable with psychotherapy in personal contact than those who have less patients treated via internet was confirmed. The hypothesis about the influence of age for the subjective assessment of the comparability of psychotherapy via internet and psychotherapy in personal contact must be rejected.

For RQ3, our evaluation showed a positive correlation between the number of patients of those therapists who began to use telephone for therapy during COVID-19 and a more positive actual experience compared to previous expectations regarding psychotherapy via telephone. 

Referring to RQ4, the hypotheses that younger psychotherapists evaluate the actual experience of psychotherapy via internet more positively (compared to previous expectations) than older psychotherapists was rejected. However, it turned out that the number of patients of those therapists who began to use internet for therapy during COVID-19 correlated with more positive actual experiences compared to previous expectations. 

In addition, for RQ5, a positive correlation was found between the perceived comparability of psychotherapy via internet and the extent to which therapists from Austria feel informed. Age or gender did not influence any of our dependent variables. Feeling informed, did not differ between therapists from Austria treating and those not treating patients via internet.

As a result, we reject the hypothesis that age plays a role as an influencing factor. It turned out that the factor knowledge is related to the perceived comparability of psychotherapy via internet. Regarding knowledge, we did not find an effect of knowledge (feeling informed) on the use of internet for psychotherapy and therefore reject our fifth hypothesis. Our third and fourth hypothesis could be validated in sum. 

Altogether, our results can be well integrated into previous research. In contrast to some other studies [19,34] in our sample age and gender did not play a role with regard to attitudes towards and experiences with psychotherapy via internet/telephone. On the other hand, like in the literature [13,19,22], the psychotherapists’ experience (in our case, the number of patients) with digital media in the context of psychotherapy is associated as an important influencing factor for the use and attitude towards psychotherapy via internet and telephone. As a possible explanation for the differences, we suspect that no standardized survey instruments were used across the studies. Since this is a cross-sectional study, we cannot disentangle the causal relationships driving the found associations. It is equally plausible to argue that people with a more positive attitude towards a particular medium being prepared to use it more as it is to consider the reverse, namely that more familiarity with a medium and how to deliver effective psychotherapy that way, informs the attituded. A possible problem could be that that everything was judged retrospectively, therefore it is possible that the participants formed one holistic judgement including initial skepticism that was overcome by the actual use of the technological options. 

The major limitation of the study is the cross-sectional design: multiple measurement points to investigate the RQs longitudinally would have more advantages. Due to the cross-sectional nature of our study, no causal conclusions can be drawn from the results. Moreover, the Austrian survey was in the early weeks of the COVID-19 lockdown and the German survey took place when restrictions were not so strong anymore and there had been some time to work out the technological, legal and reimbursement issues associated with the different routes of delivery. Moreover, no psychometrically established scales were used. For the future, it would be a good idea to construct uniform standardized survey instruments for questioning experiences and comparability of psychotherapy via telehealth. Additionally, it can be assumed that bias could have occurred and that distortions in the answers occurred due to the study design (retrospective survey). In order to prevent bias, longitudinal studies with several measurement points should be conducted. For future research it would be interesting to investigate in which areas of the psychotherapeutic process (for example, content, relationship formation, structure) differences between remote and face-to-face psychotherapy can be detected. 

A very important result of our study was that we could identify a positive correlation between feeling informed and the perception of comparability of psychotherapy via internet and psychotherapy in personal contact in Austria. This highlights the importance of providing good guidelines for the use of digital media in the context of psychotherapy and of developing training and education concepts in this context. Knowledge and understanding of remote treatment are also influencing factors regarding patients attitudes and acceptance towards telephone treatment. Addressing to these two factors, with special focus on existing prejudices and preconceptions about telephone treatment, is key to improve the acceptability with telephone treatment [40,41]. Previous research shows that patients also benefit from telemedicine. The most frequently cited factors from the patients′ perspective were, for example, user-friendliness, low costs, improved communication, and shorter travel times [42,43]. Important factors influencing patient satisfaction include their overall understanding of telehealth [41], quality of care received, and telehealth′s convenience [44]. It is therefore important to support this digital progress and to further establish telemedicine in the health care system. In this context, attention should be paid also to the aspects of the process, the contact details of psychotherapists, any risks that may arise from the use of e-mental health (e.g., the occurrence of technical difficulties) and the precautions taken to ensure confidentiality and data protection. Particular emphasis should be placed on the observance of ethical standards throughout the entire process of developing and using e-mental health [45]. Future research should address this very important topic.

## Figures and Tables

**Table 1:** Demographic characteristics of the sample and drop-out analysis.

Characteristics	Sample (*n* = 237)	Included (*n* = 190)	Excluded (*n* = 47)	Statistics
**Gender, *n* (%)**				
Female	185 (78.1)	148 (77.9)	37 (78.72)	*χ*^2^ (1) = 0.02; *p* = 0.902
Male	52 (21.9)	42 (22.1)	10 (21.3)	
**Age in years, mean (SD)**	48.19 (10.24)	47.67 (10.15)	50.30 (10.42)	*T* (235) = 1.58; *p* = 0.115
**Country, *n* (%)**				
Austria	151 (63.7)	120 (63.2)	31 (66.0)	*χ*^2^ (1) = 0.13; *p* = 0.721
Germany	86 (36.3)	70 (36.8)	16 (34.0)	
**Setting, *n* (%)**				
Independent	192 (81.0)	152 (80.0)	40 (85.1)	*χ*^2^ (2) = 1.52; *p* = 0.467
Employed	7 (3.0)	5 (2.6)	2 (4.3)	
Both	38 (16.0)	33 (17.4)	5 (10.6)	
**Registered in years (SD)**	10.13 (7.97)	10.11 (7.99)	10.21 (8.00)	*T* (232) = 0.08; *p* = 0.939
**Patient groups, *n* (%)**				
Adults	87 (36.7)	73 (38.3)	14 (29.8)	*χ*^2^ (2) = 1.23; *p* = 0.541
Children	44 (18.6)	34 (17.9)	10 (21.3)	
Both	106 (44.7)	83 (43.7)	23 (48.9)	

**Table 2:** Results of correlational analyses.

	Age	Sex	Number of Patients Internet	Number of Patients Telephone	Feel Informed
Actual experiences with internet compared to previous expectation	0.107	−0.133	0.341 **	-	0.181
Actual experiences with telephone compared to previous expectation	0.001	−0.059	-	0.238 *	-
Comparability of internet with face-to-face	0.018	−0.095	0.198 *	-	0.242 *
Comparability of telephone to face-to-face	0.145	−0.007	-	0.240 *	-

** The correlation is significant at the level of 0.01 (2-sided). * The correlation is significant at the level of 0.05 (2-sided).
